# Neurotrophin Crosstalk in the Etiology and Treatment of Neuropsychiatric and Neurodegenerative Disease

**DOI:** 10.3389/fnmol.2022.932497

**Published:** 2022-07-15

**Authors:** Rajeev Joshi, Stephen R. J. Salton

**Affiliations:** ^1^Nash Family Department of Neuroscience, Icahn School of Medicine at Mount Sinai, New York, NY, United States; ^2^Graduate School of Biomedical Sciences, Icahn School of Medicine at Mount Sinai, New York, NY, United States; ^3^Icahn School of Medicine at Mount Sinai, Friedman Brain Institute, New York, NY, United States; ^4^Brookdale Department of Geriatrics and Palliative Medicine, Icahn School of Medicine at Mount Sinai, New York, NY, United States

**Keywords:** Alzheimer’s disease, TLQP-62, TrkB, VGF, MDD (major depressive disorder), BDNF (brain derived neurotrophic factor), VEGF – vascular endothelial growth factor, ADNP (activity dependent neuroprotective protein)

## Abstract

This article reviews the current progress in our understanding of the mechanisms by which growth factors, including brain-derived neurotrophic factor (BDNF) and vascular endothelial growth factor (VEGF), and select neurotrophin-regulated gene products, such as VGF (non-acronymic) and VGF-derived neuropeptides, function in the central nervous system (CNS) to modulate neuropsychiatric and neurodegenerative disorders, with a discussion of the possible therapeutic applications of these growth factors to major depressive disorder (MDD) and Alzheimer’s disease (AD). BDNF and VEGF levels are generally decreased regionally in the brains of MDD subjects and in preclinical animal models of depression, changes that are associated with neuronal atrophy and reduced neurogenesis, and are reversed by conventional monoaminergic and novel ketamine-like antidepressants. Downstream of neurotrophins and their receptors, VGF was identified as a nerve growth factor (NGF)- and BDNF-inducible secreted protein and neuropeptide precursor that is produced and trafficked throughout the CNS, where its expression is greatly influenced by neuronal activity and exercise, and where several VGF-derived peptides modulate neuronal activity, function, proliferation, differentiation, and survival. Moreover, levels of VGF are reduced in the CSF of AD subjects, where it has been repetitively identified as a disease biomarker, and in the hippocampi of subjects with MDD, suggesting possible shared mechanisms by which reduced levels of VGF and other proteins that are similarly regulated by neurotrophin signaling pathways contribute to and potentially drive the pathogenesis and progression of co-morbid neuropsychiatric and neurodegenerative disorders, particularly MDD and AD, opening possible therapeutic windows.

## Introduction

Major depressive disorder (MDD) is among the most prevalent psychiatric disorders across the world with a high economic and psychological burden. According to the World Mental Health (WMH) survey (Otte et al., 2016), depression has an average annual prevalence of 6%, with 20% of the world’s population fulfilling the criteria for MDD once in their lifetime, and the economic burden of depression in the United States estimated to be $83.1 billion in 2000 (Cohen and Sclar, 2012) and increased from $236.6 billion to $326.2 billion between 2010 and 2018 (Greenberg et al., 2021). Females are at a two-fold higher risk than males, after puberty, of developing MDD, although it can affect either sex at any age, with a median age of onset of 25 years, peaking from mid-late adolescence until the early 40 s (Otte et al., 2016).

The oldest and still currently employed treatment option for MDD is monoamine therapy, based on the hypothesized interaction of monoamines norepinephrine and serotonin that modulates this condition (Hirschfeld, 2000). Widely used MDD therapeutics include selective serotonin and norepinephrine uptake inhibitors (SSRIs and SNRIs), which increase synaptic serotonin or norepinephrine levels, respectively, resembling treatment with monoamine oxidase inhibitors (MAOIs), although SSRIs and SNRIs are more widely used and have significantly lower rates of relapse than MAOI therapy (Clevenger et al., 2018). Although the treatment rates for MDD have increased, and different treatments have been introduced, patient response rates have remained relatively low (Cohen and Sclar, 2012), and treatment-resistant depression is common, encouraging the search for novel antidepressant treatment strategies that result from a better understanding of disease pathophysiology.

Dysregulation and loss of neuronal density in the hippocampus during chronic stress are associated with MDD and may be responsible for symptoms, such as depressed mood, anhedonia, and low energy or fatigue, which are thought to result from induced changes in intracellular signaling, gene modification, neuronal function, and connectivity, in the limbic brain regions that regulate mood and drive (Wong and Licinio, 2001; Duric and Duman, 2013). Chronic stress, aging, and depressive mood reduce neurogenesis in the hippocampus and increase neuronal apoptosis in the rodent cerebral cortex (Bachis et al., 2008). Currently used antidepressants generally positively affect neurotransmitter signaling in the limbic system, rescuing neuronal loss and the deficits in neurogenesis and intracellular signaling that are associated with anhedonia and low mood (Warner-Schmidt and Duman, 2006; Rantamaki and Castren, 2008; Duric and Duman, 2013). This suggests a potentially shared mechanism for antidepressant actions that neurotrophic growth factors and downstream proteins they regulate could therapeutically target. Although not explained by common genetic variants (Gibson et al., 2017), major depression is frequently comorbid with Alzheimer’s disease (AD), and both conditions have significant heritable components and shared dysfunction in neuroinflammation, oxidative stress, cellular signaling, and neurotransmission (Martin-Sanchez et al., 2021). Given the evidence of common pathways and associated changes in MDD and AD, genome-wide association and multiscale causal network studies have sought to uncover molecular signatures and genetic variations shared between AD and MDD, indicative of potential comorbidity (Sullivan et al., 2012; Porcelli et al., 2013; Beckmann et al., 2020; Martin-Sanchez et al., 2021).

## Neurotrophins and Depression

**FIGURE 1:**
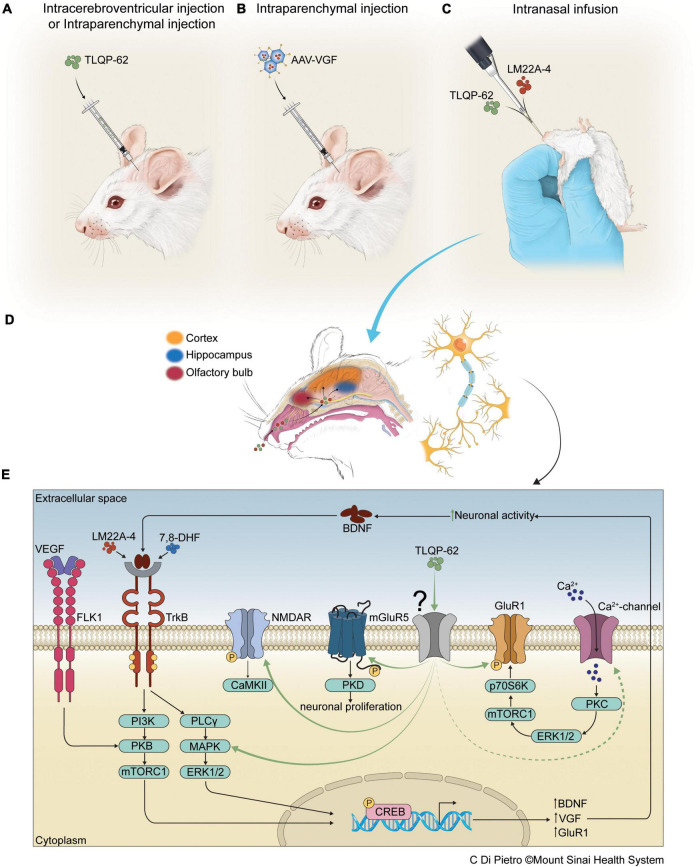
Antidepressant efficacy of therapeutics, delivered to CNS *via* intracortical, intrahippocampal, intracerebroventricular, and intranasal routes, that target neurotrophin signaling pathways. Panels **(A,B)** depict intracerebroventricular (icv), intracortical, or intrahippocampal infusions of neuropeptide TLQP-62 or AAV-VGF into rodent brain, while panel **(C)** depicts intranasal administration of small molecule agonists, including the TrkB agonist LM22A-4, and neuropeptides, such as TLQP-62, for delivery of pharmacotherapeutics to the CNS. Proposed diffusion pathways of agents delivered intranasally, bypassing the blood-brain-barrier (BBB), for distal delivery *via* the CSF and the perivascular spaces around cranial nerves, to the various brain regions shown in panel **(D)**. In panel **(E)**, the antidepressant-like actions of TLQP-62 are shown to be mediated through an unknown receptor (in gray), leading to activation of known glutamatergic signaling pathways, including NMDAR and mGluR5 (Thakker-Varia et al., 2014), the BDNF/TrkB signaling cascade (Jiang et al., 2019a), and calcium channels which activate GluR1 (Jiang et al., 2019a). Other BDNF mimetic/agonists, including LM22A-4 (Fletcher et al., 2021) and 7,8-DHF (Wurzelmann et al., 2017), have shown antidepressant efficacy *via* TrkB activation. Additionally, vascular endothelial growth factor (VEGF) is shown to induce antidepressant effects *via* PI3K/PKB/mTORC1 pathways that activate CREB. Activated CREB increases BDNF and VGF transcription, leading to increased BDNF and VGF translation and secretion, and stimulation of BDNF/TrkB signaling – an autoregulatory loop.

Neurotrophic factors and their signaling pathways have been proposed to be major targets responsible for the efficacy of antidepressant therapy (Duman, 2004, 2005; Schmidt et al., 2008). These growth factors regulate peripheral nervous system (PNS) and CNS neuronal generation, proliferation, differentiation, and survival, and include the classical neurotrophin family, specifically nerve growth factor (NGF), brain-derived neurotrophic factor (BDNF), neurotrophin-3 (NT-3), neurotrophin-4 (NT-4), and cytokine family members, such as vascular endothelial growth factor (VEGF) (Nagahara and Tuszynski, 2011) (Figure 1).

### Brain-Derived Neurotrophic Factor Function in Major Depressive Disorder

In addition to their major role in neuronal survival and function during development and in adulthood, growth factors, such as BDNF exert important pro-survival and functional effects in models of neurological, neuropsychiatric, and neurodegenerative disorders (Nagahara and Tuszynski, 2011), mediated in many cases through changes in gene expression (Lindholm et al., 1994). Studies of postmortem human brain tissues have revealed reduced BDNF mRNA and protein levels in the limbic regions of the brain, including the hippocampus, prefrontal cortex (PFC), and amygdala of depressed patients and suicide subjects (Dwivedi et al., 2003; Karege et al., 2005). In animal models, a significant reduction of BDNF expression in the dentate gyrus of the hippocampus in rats exposed to chronic restraint stress has been reported (Smith et al., 1995; Nowacka and Obuchowicz, 2013). However, in the ventral tegmental area (VTA)-nucleus acumbens (NAc) limbic projections, increased BDNF release is associated with increased susceptibility to pro-depressive behavior (Berton et al., 2006; Shirayama et al., 2015), while in models of chronic and sub-chronic variable stress (SCVS), reduced BDNF expression in hippocampal dentate gyrus and NAc was reported (Rasmusson et al., 2002; Nowacka and Obuchowicz, 2013; Brancato et al., 2017).

The human *BDNF* Val66Met gene polymorphism is the most widely studied variation with a single nucleotide polymorphism (SNP) at nucleotide 196 leading to a substitution of methionine (Met) for valine (Val) at codon 66, Val66Met, which interferes with BDNF trafficking, sorting, and secretion, and is associated with cognitive deficits (Egan et al., 2003; Chen et al., 2008; Chiaruttini et al., 2009; Nowacka and Obuchowicz, 2013). Meta-analysis pooling of gene association studies of human subjects with the Val66Met BDNF SNP showed an association with depression in late life only. However, the Val66Met SNP (1) failed to predict MDD risk, (2) was correlated with MDD in males only, and (McCullough et al., 2000) had increased chronicity of MDD with no association with disease recurrence (Verhagen et al., 2010; Gyekis et al., 2013; Lee et al., 2013; Tsang et al., 2017; Youssef et al., 2018). Meta-analysis further indicated that the BDNF Met allele carriers had an increased antidepressant response compared with Val/Val homozygotes in the Asian population (Zou et al., 2010; Kishi et al., 2017), whereas, another study revealed varied responses to a different conventional antidepressant in Val/Val homozygotes and Met carriers in depressed Caucasian subjects (Colle et al., 2015b). Perturbed BDNF expression has therefore been linked to depressive disorders (Alder et al., 2003; Dwivedi et al., 2003; Duman, 2004; Chen et al., 2008; Colle et al., 2015a; Bai et al., 2016; Jiang et al., 2019b).

Several classes of antidepressant drugs, including MAOIs, SSRIs, tricyclic antidepressants (TCAs), and SNRIs, increase BDNF expression in the brain in healthy rodents. Rapid-acting antidepressants, such as ketamine increase TrkB phosphorylation in the rat hippocampus (Garcia et al., 2008). Utilizing selective transcription and translation inhibitors, the antidepressant efficacy of ketamine and other NMDA receptor antagonists was found to require a robust increase in BDNF translation but not transcription (Garcia et al., 2008; Bjorkholm and Monteggia, 2016). BDNF protein levels are also differentially expressed in the brain following acute and chronic antidepressant treatment. Repeated application of electroconvulsive shock (ECS), but not a single application, produced 40–100% increases in BDNF levels in the hippocampus, cortex, amygdala, and brainstem; however, chronic but not acute treatment with antidepressants, such as desipramine (TCA), fluoxetine (SSRI), and phenelzine (MAOI), increased BDNF protein levels in the frontal cortex (10–30%) but not in the hippocampus, olfactory bulb, amygdala and brainstem (Balu et al., 2008).

Brain-derived neurotrophic factor (BDNF), however, lacks suitable pharmacokinetics for systemic administration due to its short plasma half-life and poor blood brain barrier (BBB) penetration. Consequently, BDNF mimetics with better pharmacokinetics have been administered that target the tropomyosin-like receptor kinase B (TrkB) receptor for BDNF. The TrkB agonist LM22A has been intranasally administered to rodents (Massa et al., 2010), showing promising results by activating TrkB, PI3K-Akt, and Ras-MAPK pathways, which play a critical role in MDD (Reichardt, 2006; Dietz et al., 2009; Duman and Aghajanian, 2012; Lu et al., 2014). Interestingly, the levels of BDNF and its receptor TrkB are inversely related; increased TrkB expression accompanies decreased BDNF expression in a repeated immobilization stress model and the forced swim test (Nibuya et al., 1999; Shi et al., 2010). Expression and activation of brain BDNF/TrkB pathways are also highly region- and circuit-specific. For example, a single bilateral infusion of TrkB agonist 7,8-Dihydroxyflavone (7,8-DHF), but not the TrkB antagonist ANA-12, into the infralimbic part of the medial prefrontal cortex (mPFC), dentate gyrus (Levi et al., 2004) and CA3 region exerted antidepressant effects in learned helplessness (LH) rat model, while the effect was absent following infusion of 7,8-DHF into the prelimbic region of the PFC (Shirayama et al., 2015; Zhang et al., 2015). In contrast, bilateral infusion of ANA-12 but not 7,8-DHF, into NAc exerted an antidepressant-like effect in LH rats (Shirayama et al., 2015). Furthermore, intraperitoneal (IP) injection of 7,8-DHF or ANA-12 also led to antidepressant and pro-depressant responses, respectively (Zhang et al., 2015). While BDNF/TrkB activation has antidepressant efficacy, proBDNF binding to its receptor p75NTR has the opposite effect; proBDNF/p75NTR signaling is upregulated, and BDNF/TrkB signaling is downregulated by chronic mild unpredictable stress in rats (Bai et al., 2016).

### The Role of Vascular Endothelial Growth Factor in Major Depressive Disorder

The antidepressant efficacies of ketamine and desipramine are reduced by gene ablation of *BDNF* and vascular endothelial growth factor (*VEGF*), respectively (Warner-Schmidt and Duman, 2006; Lin et al., 2015). VEGF is a potent mitogen and survival factor for endothelial cells and neurons, and additionally modulates synaptic transmission in the adult hippocampus and subventricular zone (Fabel et al., 2003; McCloskey et al., 2005), neuroprotection (Feng et al., 2011), and hippocampal neurogenesis (Fournier and Duman, 2012), consistent with a potential role in MDD. The VEGF family consists of six members: VEGF (A-E) and placental-derived growth factor (P1DF). VEGF-A is spliced into four variants, with VEGF_120_ and VEGF_164_ known to be the predominant brain variants (Warner-Schmidt and Duman, 2006). Furthermore, in addition to its regulation by antidepressants, VEGF is also modulated by exercise, a potent stimulator of neurogenesis and neurotrophic growth factor expression (Fabel et al., 2003; Warner-Schmidt and Duman, 2006; Ploughman, 2008). Nevertheless, some studies have found no correlation between BDNF and VEGF levels with aerobic exercise in depressed patients (Krogh et al., 2014). Currently, exercise is thought to affect executive functioning by (1) increasing oxygen saturation and angiogenesis in brain areas crucial for task performance, (2) increasing neurotransmitter levels, and (McCullough et al., 2000) upregulating neurotrophic growth factors, such as BDNF (Duman and Monteggia, 2006; Ploughman, 2008). Given the evidence that exercise stimulates brain angiogenesis and increases oxygen saturation, implicating a role for VEGF, it is interesting that Fabel et al. (2003) reported that blockade of peripheral VEGF signaling in a rat exercise model reduced CNS neurogenesis, and that removal of VEGF-antagonist increased neurogenesis, suggesting that exercise-induced neurogenesis requires peripheral VEGF signaling. The biological effects of VEGF are transduced by two receptor tyrosine kinases, fetal liver kinase (Flk-1 or VEGFR2) and fms-like tyrosine kinase (Flt-1), and by a family of putative coreceptors called neuropilins (NRPs) (Warner-Schmidt and Duman, 2006). Therefore, pharmacological modulation of VEGF receptors could have efficacy in MDD, consistent with the recent finding that neuronal VEGF-VEGFR2 (Flk-1) pathways play a key role in the rapid antidepressant actions of ketamine (Deyama et al., 2019b). Recent studies further demonstrate that antidepressant actions of VEGF in mPFC are blocked by neutralizing anti-BDNF antiserum, while antidepressant actions of BDNF in mPFC are blocked by VEGF ablation or neutralizing anti-VEGF antiserum (Deyama et al., 2019a; Duman et al., 2021). VEGF, therefore, functions in complex interplay with BDNF in the mechanism of action of ketamine. Nevertheless, the neuropathological effects of VEGF in MDD and other neuropsychiatric conditions, including schizophrenia require additional investigation (Tsang et al., 2017; Pu et al., 2020).

### VGF Functions Downstream of Neurotrophin Signaling Pathways to Regulate Major Depressive Disorder

Brain-derived neurotrophic factor (BDNF) and TrkB play crucial roles in cognition, memory, and depression (Duman and Monteggia, 2006; Rantamaki and Castren, 2008; Nagahara and Tuszynski, 2011), driven at least in part by downstream effectors, including VGF (non-acronymic) (Bonni et al., 1995; Alder et al., 2003). VGF is a neurosecretory protein that was first identified (Levi et al., 1985) as a nerve growth factor (NGF)-regulated transcript in rat pheochromocytoma cells (PC12), and was independently identified by the Wagner and Salton labs (Cho et al., 1989; Salton et al., 1991), and belongs to the granin family (Levi et al., 1985; Salton et al., 2000; Bartolomucci et al., 2011). VGF mRNA is widely expressed in the PNS and CNS, abundantly in the hypothalamus, consistent with known function in metabolic regulation and energy balance (Snyder and Salton, 1998; Hahm et al., 1999, 2002), and in the hippocampus, in line with important roles for VGF in memory and depression-like behavior (Bozdagi et al., 2008; Lin et al., 2015, 2021; Li et al., 2017; Jiang et al., 2019a). VGF undergoes proteolytic cleavage by prohormone convertases and proteases in the regulated secretory pathway to produce at least 12 VGF-derived peptides (Levi et al., 2004; Quinn et al., 2021). VGF-derived peptides have been reported in several CSF proteomic studies to be potential biomarkers of neuropsychiatric and neurodegenerative disorders, including MDD and AD (Bartolomucci et al., 2010; Llano et al., 2019; van Steenoven et al., 2020; Quinn et al., 2021), consistent with possible functional roles for VGF in disease pathophysiology. Indeed, *Vgf* gene ablation impacts memory and hippocampal function (Bozdagi et al., 2008; Lin et al., 2015), and infusion of VGF-derived peptide TLQP-62 into the brain improves memory (Lin et al., 2015; Li et al., 2017). VGF expression in the hippocampus is decreased in mouse models of depression-like behavior and in postmortem samples of hippocampus and Brodmann area 25 from both medicated and unmedicated male and female patients with MDD compared to controls (Jiang et al., 2018, 2019a), while expression is increased by exercise and chronic antidepressant treatment, including fluoxetine and ketamine (Hunsberger et al., 2007; Thakker-Varia et al., 2007; Jiang et al., 2018, 2019a). VGF overexpression in mouse vmPFC *via* infusion of AAV-VGF rescued behavioral deficits induced by chronic restraint stress (Jiang et al., 2019a). Furthermore, ketamine and desipramine antidepressant efficacies were reduced by targeted VGF ablation in the hippocampus and/or vmPFC (Jiang et al., 2018, 2019a), reminiscent of the reduction in antidepressant efficacy of voluntary exercise that was observed in germline VGF-deficient mice (Hunsberger et al., 2007). Taken together, these studies support a critical role of the neurotrophin-regulated gene *Vgf* in depressive behavior and antidepressant efficacy.

### Procognitive and Antidepressant Efficacy of the VGF-Derived Peptide TLQP-62 – A Potentially Novel Therapeutic Agent for Major Depressive Disorder

Administration of the C-terminal VGF-derived peptide TLQP-62 (by convention named by its N-terminal 4 amino acids and length) to the hippocampus or vmPFC has antidepressant efficacy that was shown to be BDNF-dependent and increases pTrkB and pCREB levels (Lin et al., 2014, 2015). TLQP-62 dependence on BDNF/TrkB was determined utilizing conditional BDNF knockout mice (Jiang et al., 2018, 2019a), targeted shRNA approaches (Li et al., 2017), and BDNF/TrkB inhibitors (Lv et al., 2018). Additional experiments implicated downstream activation of mTORC1, reduced bicaudal C homolog 1 (BICC1) signaling, and increased levels of synaptic GluA1 and pGluA1, following antidepressant TLQP-62 infusion into the hippocampus or PFC (Jiang et al., 2018, 2019a,b; Lv et al., 2018). TLQP-62 infused icv (intra-cerebroventricular) or into the hippocampus improved contextual fear memory or lipopolysaccharide (LPS)-induced memory dysfunction, and either activated BDNF/TrkB pathways or was shown to require BDNF expression (Figure 1) (Lin et al., 2015; Li et al., 2017), consistent with a general mechanism underlying VGF actions in the brain. A proposed autoregulatory feedback model suggests that increased BDNF/TrkB signaling results in *Vgf* gene induction and the production of VGF-derived peptides, including procognitive and antidepressant TLQP-62, which both stimulates and requires BDNF/TrkB signaling for efficacy (Jiang et al., 2019a). Moreover, in neuronal cell cultures, pTrkB levels were significantly increased by 24 h of TLQP-62 treatment, which in addition increased neurogenesis and the proliferation of neural stem cells, and increased synaptic plasticity through actions on glutamatergic receptors, including mGluR5 and NMDA receptors (Thakker-Varia et al., 2014). Taken together, these studies suggest an important role for VGF and VGF-derived peptides as critical mediators of memory and depression-like behavior, and that novel VGF therapeutics may provide new approaches to MDD.

## Potential Mechanisms Underlying Comorbidity Between Alzheimer’s Disease and Major Depressive Disorder

**FIGURE 2:**
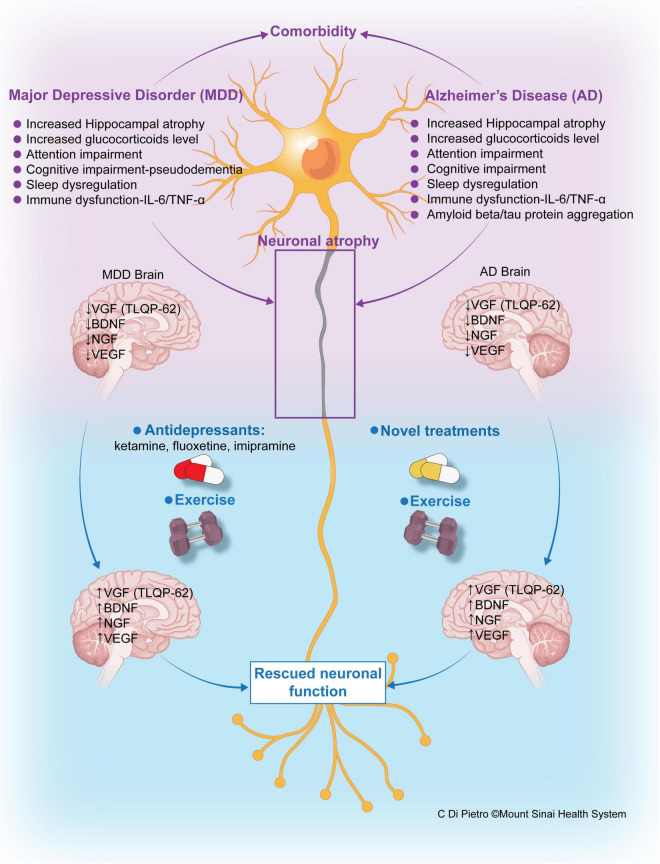
Schematic of the Comorbidity of Major Depressive Disorder (MDD) and Alzheimer’s disease (AD), disorders that share pathophysiology, disease mechanisms, and potentially treatments. MDD and AD are often comorbid, in many cases sharing a number of pathophysiological features that are summarized in this figure. Cognitive disorders are a core feature of both MDD and AD, often referred to in MDD as pseudodementia or dementia of depression. Cognitive symptoms (particularly memory) in MDD can be reversible with treatment, or more commonly can persist following improvement or remission of depressive symptoms, progressing to overt dementia and AD (Rodrigues et al., 2014; Perini et al., 2019). Immune and glucocorticoid dysfunction are common to AD and MDD, leading to neuronal atrophy, as depicted. Expression of neurotrophic growth factors, including BDNF, VEGF, and VGF, and neuropeptides, such as TLQP-62, is also reduced in both AD and MDD. Treatment with antidepressants and increased exercise increase the levels of neurotrophins in MDD, helping to rescue neuronal function. In AD, exercise and future novel therapeutics may be similarly utilized to increase levels of these neurotrophins and select downstream gene products (VGF), halting the progression of neurodegenerative disease.

The progressive neurodegenerative disorder Alzheimer’s disease (AD) is the most common form of dementia, affecting 50-75% of patients, and is reaching pandemic levels (Blennow et al., 2006), while Major Depressive Disorder (MDD) is a debilitating mental illness characterized by high prevalence and resistance to treatment. Both AD and MDD have devastating public health impacts, and comorbidity is frequent but not explained by common genetic variants (Gibson et al., 2017) and could in part be driven by overlapping pathophysiologic mechanisms and signaling networks (Figure 2). BDNF protects against neurodegeneration through signaling pathways that activate the cyclic AMP response element-binding protein (CREB) (Figure 1), increasing hippocampal neurogenesis, controlling the amyloidogenic pathway and Aβ production, and regulating downstream gene expression in the hippocampus (Matrone et al., 2008; Blurton-Jones et al., 2009; Caccamo et al., 2010). VEGF and BDNF pathways play critical, interdependent roles in the antidepressant efficacy of ketamine in MDD as noted above, while in early-onset and late-onset AD, VEGF-A expression in the brain and vasculature undergoes complex, stage-dependent dysregulation (Ali et al., 2022). In the APP/PS1 AD mouse model, intraperitoneal administration of neutralizing anti-VEGF-A antibodies ameliorated capillary stalling, normalized BBB permeability, and increased cerebral blood flow (CBF) (capillary stalling and reduced CBF are associated with cognitive impairment in AD patients) (Ali et al., 2022). On the other hand, delivery of VEGF-A to the brains of mouse AD models partially rescued vascular loss, reduced amyloid plaque load, and rescued impaired memory (Religa et al., 2013; Garcia et al., 2014) as did intraperitoneal VEGF administration (Wang et al., 2011). VEGF-A signaling in the brain may, therefore, be impaired in AD (Jefferies et al., 2013; Ali et al., 2022), much as it appears to be in MDD, suggesting a potential mechanism underlying disease comorbidity.

Among hundreds of genes involved in AD, the BDNF-regulated *VGF* gene was identified as a key network driver of AD pathogenesis and progression, and as a potential therapeutic target (Tasaki et al., 2018; Beckmann et al., 2020). VGF levels are consistently decreased in brain tissue and CSF samples of patients with AD (Ruetschi et al., 2005; Zhao et al., 2008; Bartolomucci et al., 2010; Cocco et al., 2010; Asano et al., 2011; Jahn et al., 2011; Wijte et al., 2012; Hendrickson et al., 2015; Holtta et al., 2015; Spellman et al., 2015) and postmortem brain tissue samples from depressed male and female subjects (Jiang et al., 2018, 2019a) as compared with controls. VGF levels were also reduced in patients with MCI only in those who then progressed to AD, further indicating the potential role of VGF as a biomarker in dementia (Jahn et al., 2011; Llano et al., 2019). Importantly, overexpression of VGF in preclinical 5xFAD mouse AD models, *via* germline overexpression, AAV-mediated VGF delivery, or long-term icv TLQP-62 or TLQP-21 peptide administration, reduced amyloid load, phospho-tau levels, synaptic pathology, dystrophic neurite damage, astrogliosis, microgliosis, and memory impairment, and rescued neurogenesis and LTD deficits (Beckmann et al., 2020; El Gaamouch et al., 2020). Thus, VGF is perhaps one of a limited number of proteins that are down-regulated in both AD and MDD.

The activity-dependent neuroprotective protein (ADNP), which plays a key role in neurogenesis and is essential for brain formation, is another protein that is decreased in postmortem AD brain samples. Accumulating mosaic somatic mutations in the *ADNP* and other genes in the brains of patients with AD have been proposed to contribute to disease progression, including impaired cognition, increased amyloid plaque burden, and tauopathy (Park et al., 2019; Gozes, 2021; Ivashko-Pachima et al., 2021). Low-level brain somatic mutations in glutamate and dopamine receptor signaling pathway genes have similarly been identified in neuropsychiatric diseases, specifically schizophrenia (Gozes, 2021; Kim et al., 2021). ADNP additionally functions in the pathophysiology of schizophrenia *via* a key regulatory role in autophagy, which when it is dysregulated also contributes to AD pathogenesis (Merenlender-Wagner et al., 2014, 2015; Sragovich et al., 2017). Recently identified ADNP interactions with SHANK3 (Ivashko-Pachima et al., 2022) and SIRT1 (Hadar et al., 2021) may provide critical new targets for understanding the role that ADNP plays in neuropsychiatric, neurodevelopmental, and neurodegenerative disease. Furthermore, the smallest active snippet of ADNP, an eight-amino-acid peptide NAP (NAPVSIPQ), protected against ADNP deficiencies in autism patients with Helsmoortel-Van Der Aa syndrome (Merenlender-Wagner et al., 2014; Sragovich et al., 2017; Gozes, 2021; Ivashko-Pachima et al., 2021). Therefore, therapeutic overexpression or CNS administration of these ADNP- or VGF-derived peptides have the potential to prevent the progression of comorbid neuropsychiatric and neurodegenerative diseases.

Neurotrophic growth factors and their regulated gene products, such as VGF, could regulate comorbid MDD and AD *via* shared actions on neurogenesis or synaptic plasticity. Neurogenesis is a dynamic process that is known to be regulated by various external stimuli, including physiological, pathological, and pharmacological changes, that also regulate hippocampal synaptogenesis (Duman, 2005; Thakker-Varia et al., 2014). These stimuli include exercise, enriched environment, antidepressants, and intrinsic factors that include neurotrophic growth factors, such as BDNF, VEGF, VGF, and VGF-derived neuropeptides, like TLQP-62 (Salton et al., 2000; Duman, 2005; Dranovsky and Hen, 2006; Thakker-Varia et al., 2014). Several clinical studies have linked depression with decreased hippocampal volume associated with decreased pyramidal neuronal arborization (Sapolsky, 2000; Sheline, 2000), and perhaps novel peptides like TLQP-62 could find therapeutic utility if they were able to restore neuronal loss and rescue hippocampal atrophy. However, clinical evidence is very limited, and therefore additional studies are required to understand the process of neuronal loss in patients suffering from depression (Sapolsky, 2000; Czeh and Lucassen, 2007).

In pre-clinical studies, TLQP-62 infused intrahippocampally in rodents has antidepressant efficacy, and VGF overexpression or icv peptide administration reduces AD-related phenotypes in 5xFAD mice. However, an extension of these findings to clinical trials that assess novel therapeutics in human subjects, involving either CNS gene therapy (AAV-VGF) or intranasal peptide administration, still has significant technical hurdles to overcome. In this regard, it is somewhat sobering that rescue of reduced levels of BDNF in the hippocampus and entorhinal cortex by sustained BDNF gene delivery *via* viral vectors, in subjects with AD, did not have significant clinical efficacy (Connor et al., 1997; Hock et al., 2000; Nagahara and Tuszynski, 2011), although it does encourage the search for novel, alternative therapeutics that activate these pathways and may have better clinical results.

## Author Contributions

RJ and SS wrote the manuscript. Both authors contributed to the article and approved the submitted version.

## Conflict of Interest

The authors declare that the research was conducted in the absence of any commercial or financial relationships that could be construed as a potential conflict of interest.

## Publisher’s Note

All claims expressed in this article are solely those of the authors and do not necessarily represent those of their affiliated organizations, or those of the publisher, the editors and the reviewers. Any product that may be evaluated in this article, or claim that may be made by its manufacturer, is not guaranteed or endorsed by the publisher.
